# Acotiamide and Functional Dyspepsia: A Systematic Review and Meta-Analysis

**DOI:** 10.7759/cureus.20532

**Published:** 2021-12-20

**Authors:** Dhan B Shrestha, Pravash Budhathoki, Prarthana Subedi, Manita Khadka, Prabesh Karki, Yub Raj Sedhai, Bhesh Raj Karki, Wasey Ali Yadullahi Mir

**Affiliations:** 1 Department of Internal Medicine, Mount Sinai Hospital, Chicago, USA; 2 Department of Internal Medicine, BronxCare Health System, Bronx, USA; 3 Department of Internal Medicine, Nepalese Army Institute of Health Sciences, Kathmandu, NPL; 4 Department of Internal Medicine, Virginia Commonwealth University School of Medicine, Richmond, USA; 5 Department of Internal Medicine, State University of New York Downstate Health Sciences University, New York, USA

**Keywords:** epigastric pain, prolactin, meta-analysis, acotiamide, functional dyspepsia

## Abstract

Functional dyspepsia is a common gastrointestinal disorder characterized by postprandial fullness or early satiety and epigastric burning or pain in the absence of organic disease. Acotiamide is a novel prokinetic motility drug being used in functional dyspepsia. Databases like PubMed, PubMed Central, Embase, and Scopus were searched for studies comparing the use of acotiamide and placebo for people with functional dyspepsia. Quantitative synthesis was performed using RevMan 5.4 (Cochrane, London, United Kingdom). The improvement in symptoms of functional dyspepsia after treatment was higher in people treated with acotiamide than placebo, although not statistically significant (OR, 1.48; 95% CI, 0.93 to 2.35; n = 1697; I^2^ = 59%). Among the commonly reported adverse effects, namely, raised in serum prolactin (OR 1.02, 95% CI 0.64 to 1.61; n = 1709; I^2^ = 44%), raised in alanine transaminase (OR 1.27, 95% CI 0.70 to 2.33; n = 1709; I^2^ = 0%), and raised in serum bilirubin (OR, 0.98; 95% CI, 0.52 to 1.87; I^2^ = 0%) did not differ between two groups. Acotiamide seems to be a promising agent in functional dyspepsia. However, further larger studies are needed to evaluate the role of acotiamide in functional dyspepsia.

## Introduction and background

Functional dyspepsia (FD) is a condition characterized by a group of symptoms originating from the gastroduodenal region without organic disease that readily explains the symptoms [1]. Functional dyspepsia is one of the common gastrointestinal conditions that affect almost 1/5th of the population. According to Rome IV, the condition is subdivided into three categories postprandial distress syndrome (PDS), epigastric pain syndrome (EPS), and overlap among EPS and PDS. Based on a large population-based cross-sectional study, the proportion of subgroups was 61% PDS, 18% EPS, and 21% overlapping PDS and EPS [2]. The exact cause of functional dyspepsia remains unknown. However, many factors can cause it. Previously it was known as an idiopathic condition. However, many factors like gastroenteritis, the presence of *Helicobacter pylori (H. pylori)* infection, gastric and duodenal motility disturbances have been attributed as the cause of functional dyspepsia (FD). Diagnosis of FD is made based on Rome IV criteria, where PDS is characterized by the presence of early satiety and postprandial fullness, which is severe enough to affect the regular activities of the individual or to finish a usual-size meal for ≥3 days/week in the past three months, with such history for a six-month duration.

EPS is diagnosed by bothersome pain or burning over epigastrium for ≥1 day/week in the last three months, with such history for at least six months. Common symptoms include early satiety and postprandial fullness. The evaluation is mainly done by esophagogastroduodenoscopy. Treatment modalities include reassurance, diet, acid suppression therapies, prokinetics, fundic relaxers, tricyclic antidepressants, rifaximin, and psychological therapies. FD is relapsing and remitting. Population-based studies showed that 15-20% of individuals do have lasting symptoms, even persisted in extended follow-up periods, and only 50% had resolution of their symptoms completely. There is no study showing survival statistics in FD. Acotiamide or Z-338 (acotiamide hydrochloride trihydrate) is a novel agent to treat FD. It acts as a gastric motility modulator and works with a different mechanism of action than prior agents.

Unlike conventional prokinetic agents, acotiamide has a low affinity for serotonin receptors, namely, 5-HT_2_, 5-HT_3_, and 5-HT_4_. In addition, acotiamide has low affinity to D_2_-receptors than any other prokinetic drug. It has an affinity for muscarinic receptors activities and exerts prokinetic activity [3]. Additionally, acotiamide inhibits the activity of acetylcholinesterase (AChE), which might help in prokinesis. In our review, we aimed to evaluate the role of acotiamide in functional dyspepsia.

## Review

Methods

We used Preferred Reporting Items for Systematic Reviews and Meta-Analyses (PRISMA) guidelines for the systematic review of the available literature [4]. 

Criteria for considering studies for this review

We included randomized control trials and other non-randomized comparative studies reporting on the use of acotiamide in functional dyspepsia compared to placebo. Editorials, comments, viewpoint articles, case reports with no proper data and lacking adequate data of interest were excluded. All the patients diagnosed with functional dyspepsia were included in the study. Those receiving acotiamide were put in the treatment group, and those on placebo were included in the control group. Acotiamide in various dosage forms was the intervention for the treatment group. Placebo and standard medical therapy (SMT) were taken as comparators. 

Our main aim was to determine the efficacy of acotiamide in functional dyspepsia vs. placebo and to determine the efficacy of acotiamide in treating EPS and PDS. Adverse events related to treatment for FD were taken as an additional outcome.

The main outcomes of this systemic review and meta-analysis were to assess the efficacy of acotiamide in functional dyspepsia vs. placebo and the efficacy of acotiamide in treating EPS and PDS. Additionally, we also did an analysis on adverse events of acotiamide in comparison to placebo during the treatment of FD.

Search methods for identification of studies

Two reviewers (PB and DBS) accessed electronic databases like PubMed, Pubmed Central (PMC), Scopus, and Embase using appropriate Boolean operators with no language restriction. Two other reviewers (PS and PK) screened the title and abstract of the studies using Covidence, and the conflicts were resolved by the third reviewer (MK). In comparison, full-text screening was done by MK and PS, and PK resolved the conflicts after discussion with the other two reviewers. Finally, the quality of individual articles was evaluated using the Cochrane risk-of-bias (RoB) 2.0 tool (Chochrane, London, United Kingdom) for randomized controlled trials (RCTs).

Data collection and analysis

We extracted the data onto a standardized form designed in Excel (Microsoft, Redmond, Washington). Statistical analysis was done in RevMan 5.4 (Cochrane, London, United Kingdom). We used the odds ratio (OR)/ risk ratio (RR) as appropriate to analyze data. The I² test measured heterogeneity. The random/ fixed model was used as per heterogeneity.

This meta-analysis was done based on the Preferred Reporting Items for Systematic Reviews and Meta-Analyses (PRISMA) guideline. In addition, the study protocol is registered in the International prospective register of systematic reviews (CRD42021228304).

Selection of studies

We included randomized control trials and other non-randomized comparative studies reporting on the use of acotiamide in functional dyspepsia compared to placebo. We excluded studies that were editorials, comments, viewpoint articles, case reports with no proper data and lacking adequate data of interest.

Data extraction and management

Data extraction from selected studies was done in accordance with the pre-drafted review protocol. First, two authors individually screened published papers based on inclusion and exclusion criteria and screened the title and abstract using Covidence. Then, discrepancies were resolved by mutual consent from the third author, and data were extracted onto a standardized form designed in Microsoft Excel 2013.

**Figure 1:**
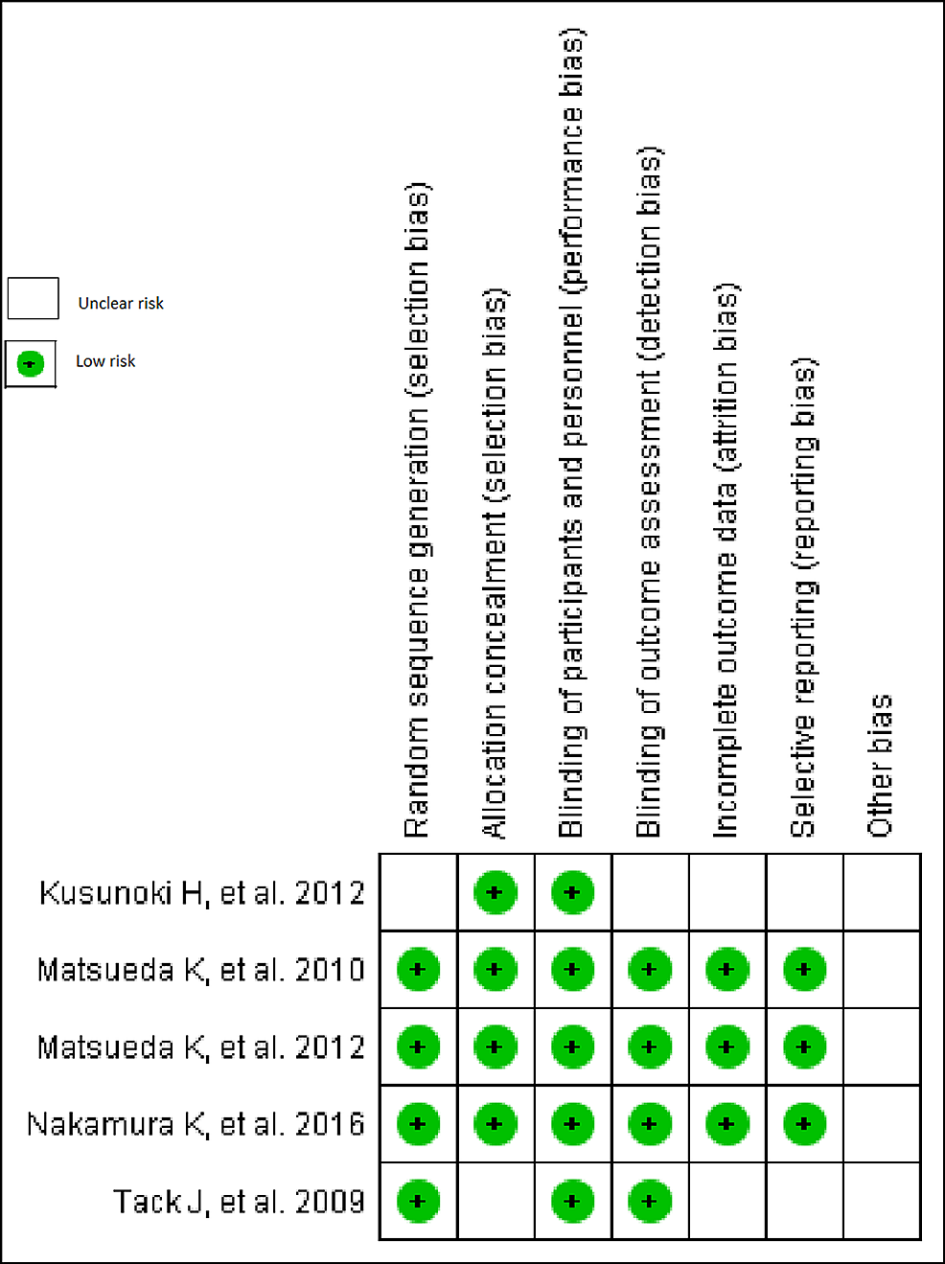
Risk of-bias-assessment Bias summary of five included studies [6-10]

All articles included in the review were assessed using the Cochrane risk-of-bias (RoB) [5]. Heterogeneity was measured by the I² test by using the Cochrane Handbook for Systematic Reviews of Interventions. Reporting bias of the individual included papers was checked by prefixed reporting of the outcome. Qualitative analysis among selected studies was presented in the table. The quantitative analysis was done in RevMan. Outcome variables were pooled using an odds ratio. Sensitivity analysis was done where required. The random/ fixed model was used for assessing heterogeneity. Figure 1 depicts the risk of bias assessment.

Results

**Figure 2:**
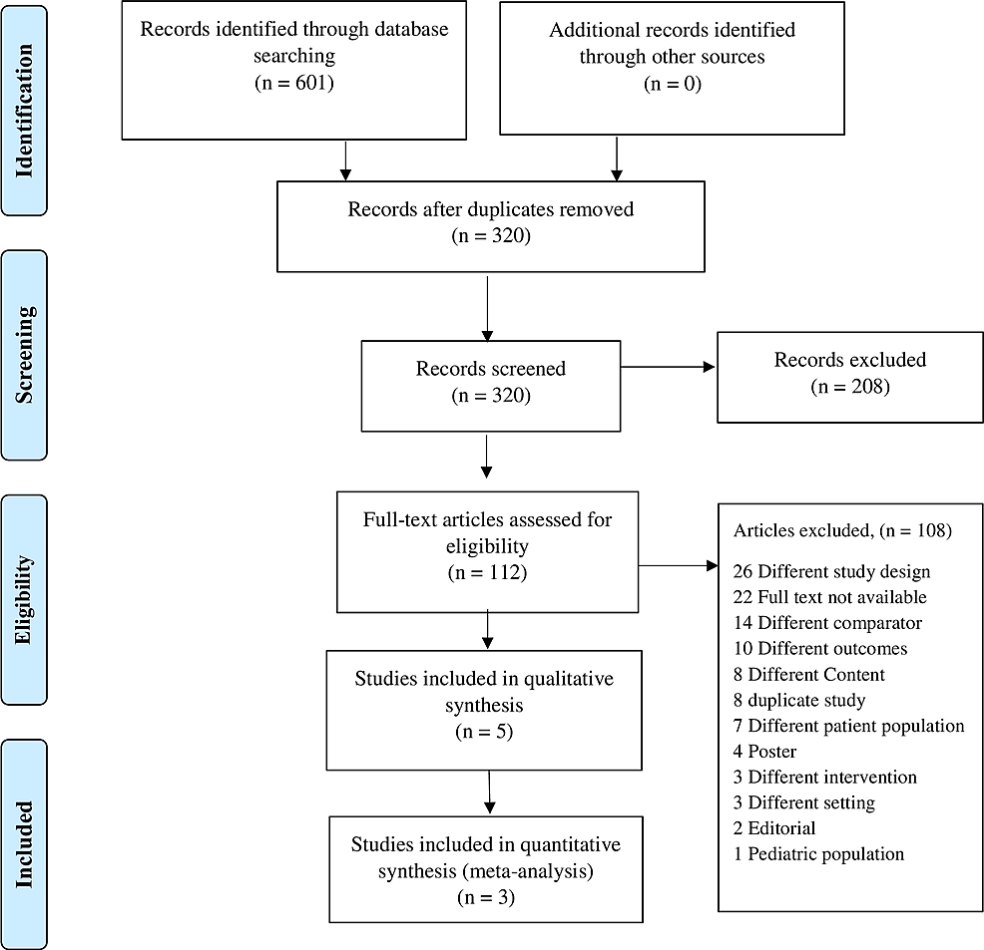
PRISMA flow diagram

After an initial search among 601 studies imported from the database, 320 studies were screened after removing 281 duplicates. Out of the 320 studies, 112 studies were considered for assessment of eligibility. After excluding unsuitable articles based on various exclusion criteria during the full-text review, five studies were included for qualitative and three in quantitative synthesis (Figure 2). 

The basic details of the included studies are given in Table 1.

**Table 1:** Basic details of the included studies FD - functional dyspepsia, IBS - irritable bowel syndrome, PDS - postprandial distress syndrome

ID	Country	Study design	Inclusion criteria	Exclusion criteria
Tack J, et al. [6] 2009	8 European countries	Randomized, double‐blind, placebo‐controlled, parallel-group study	Patients aged 18-65 presenting with FD symptoms, as defined by the Rome II classification; with dyspepsia for ≥ 12 weeks in the last one year	Subjects with predominant heartburn or symptoms of irritable bowel syndrome (IBS), any significant lesions in endoscopic findings, any severe organic disease in history or major abdominal surgery, psychiatric disorder, drugs affecting gastric secretion and gastrointestinal motility, and drugs provoking upper digestive lesions.
Matsueda K, et al. [7] 2011	Japan	Multicenter, randomized, double-blind, placebo-controlled, parallel-group, phase III trial	Patients diagnosed with FD aged 20-64 with FD-PDS abased on Rome III	Patients with any changes in esophagus, duodenum, or stomach on endoscopy, with heartburn in last 12 weeks, and patients with IBS
Nakamura K, et al. [8] 2016	Japan	Randomized, double-blind, placebo-controlled, parallel-group study	Outpatients, age ≥ 20 years, endoscopy in last one year with organic disease, not under any prokinetics, acid suppressants, muco-protective agents, antidepressants, anxiolytics, hypnotics, or antipsychotics	Eradication of *Helicobacter pylori* in last six months or currently taking regimen for eradication or hypersensitivity to acotiamide in past.
Kusunoki H, et al. [9] 2012	Japan	A randomized, double-blind, placebo-controlled, parallel study	Patients aged 20-70 years and diagnosed as having FD according to Rome II criteria.	Patients with a prior diagnosis of diabetes mellitus, prior gastrointestinal surgery, or under any medications altering gastric motility
Matsueda K, et al. [10] 2010	Japan	Randomized, double-blind, placebo-controlled, parallel-group, comparative studies	Patients aged 20-79 had FD as defined by Rome II guideline with normal endoscopic findings.	If heartburn presents as the most bothersome symptom. Patients with serious/ malignant disease, abusing drug/ alcohol, and severe ECG abnormality at rest and clinical laboratory examination. Under ulcer protective medication, antacids, prokinesis, non-steroidal anti-inflammatory drugs, or antidepressants. Women who are pregnant/breastfeeding

The narrative summary of the four included studies is presented in Table 2.

**Table 2:** The narrative summary of 4 included studies C - control, T - treatment, ALT - alanine transaminase, TDS - three times a day

Study ID	Population			Intervention	Comparison	Outcome	Side-effects (increased)		
	Participants	Age (mean)	Sex (Male/Female)				Serum ALT	Serum prolactin	Serum bilirubin
Tack J, et al. [6] 2009	T:56 (T1: 21, T2: 16, T3: 19)	T (T1:41.6; T2:42.1; T3:36.3)		Acotiamide 50mg-T1, 100mg-T2, 300mg-T3	Placebo	No significant effect on the sense of distension and gastric emptying. For accommodation to meals, 300 mg acotiamide was better comparing with placebo (p: 0.024).			
	C:15	C:49.1							
Matsueda K, et al [7] 2011	T: 450	T:37.6±10.7	T: 176/274	Acotiamide 100mg TDS	Placebo	Improvement T: 235/450	T:18/450	T: 21/450	T:19/450
	C: 442	C:37.1±9.9	C:187/255			C: 154/442	C: 17/442	C:30/442	C:18/442
Nakamura K, et al. [8] 2016	T:22	T:50.6 ± 16.5	T:6/16	Acotiamide 100 mg	Placebo	Gastric accommodation T: pre 29.6 ± 14.8 %, post 35.1 ± 13.4 %			
	C:24	C: 59.0 ± 16.5	C:6/18			C: pre 38.5 ± 15.1 %, post 35.6 ± 15.0 %			
Kusunoki H, et al. [9] 2012	T:19	T: 40.3±13.2	T:6/ 13	Acotiamide 100 mg TDS	Placebo	Improvement; T: 6/19	T:1/21	T:2/21	T:0/21
	C: 18	C:40.6±13	C:7/11			C: 3/18	C:0/21	C:1/21	C:1/21
Matsueda K, et al. [10] 2010; Study 1	T:210( T1:104, T2: 106)	T1: 37.5±11.5, T2: 38.6±12.8	T1: 50/54, T2:39/67	Acotiamide T1: 100mg, T2:300mg	Placebo	Improvement: T(T1+T2) :169/210	T(T1+T2) :5/214	T(T1+T2) :15/214	
	C: 107	C: 37.3 ± 10.2	C: 50/57			C:80/107	C:1/107	C:4/107	
Matsueda K, et al. [10] 2010; Study 2	T:339 (T1: 115, T2: 108, T3: 116)	T1: 38.9±12.4, T2:39.1 ±12.4, T3: 40.6±14.2	T1:41/74, T2: 38/70, T3: 42/74	Acotiamide T1: 50 mg, T2: 100mg, T3:300mg	Placebo	Improvement: T(T1+T2+T3) :289/339	T(T1+T2+T3) :4/342	T(T1+T2+T3) :12/342	
	C: 112	C:38.0±13.1	C: 37/75			C:98/112	C:0/112	C:1/112	

Among five studies selected for qualitative review, only three reported comparable data and were included in quantitative analysis.

Efficacy Outcome

**Figure 3:**
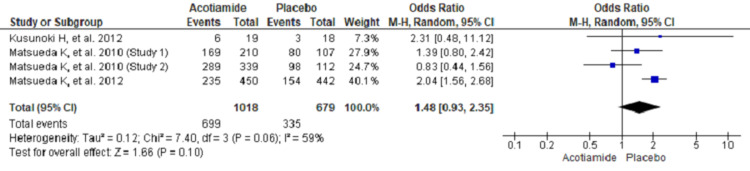
Forest plot showing improvement in symptoms of functional dyspepsia using a random-effect model Only three studies reported symptomatic improvement [7,9,10].

Pooling of the data using random-effect model showed improvement in symptoms of functional dyspepsia after treatment was higher in the treatment group (acotiamide) compared with placebo though it could not reach statistical significance (OR, 1.48; 95% CI, 0.93 to 2.35; n = 1697; I^2^ = 59%) (Figure 3).

Adverse Outcomes

**Figure 4:**
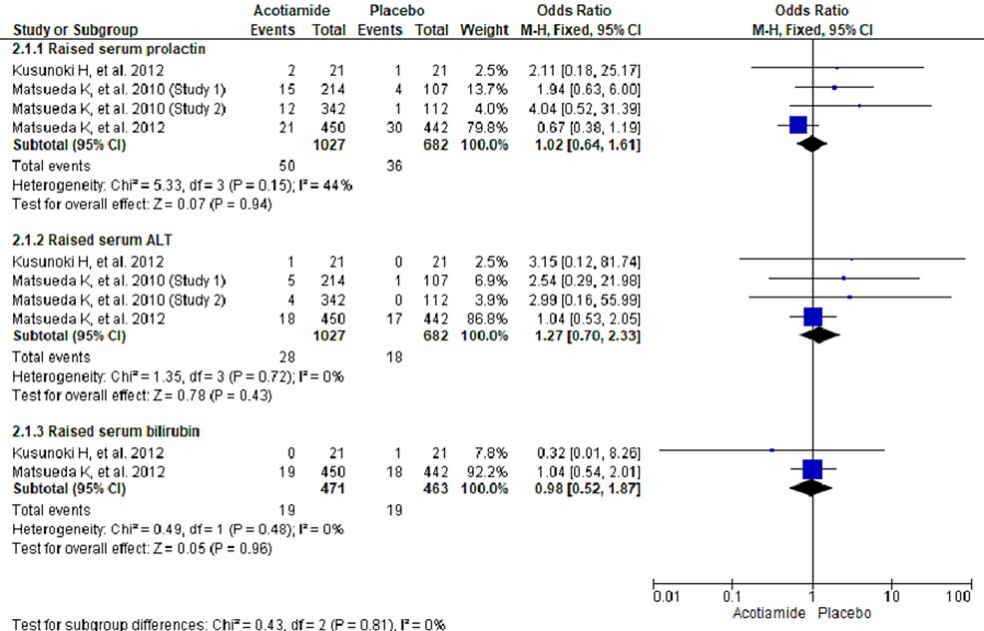
Forest plot showing adverse laboratory parameters using fixed effect model Only three studies reported adverse laboratory outcomes [7,9,10].

There were no significant differences in reported adverse effects between two groups for rise in serum prolactin (OR 1.02, 95% CI 0.64 to 1.61; n = 1709; I^2^ = 44%), rise in alanine transaminase (ALT) (OR 1.27, 95% CI 0.70 to 2.33; n = 1709; I^2^ = 0%), rise in serum bilirubin (OR, 0.98; 95% CI, 0.52 to 1.87; I 2 = 0%) (Figure 4).

Discussion

Our meta-analysis is the most comprehensive meta-analysis to compare acotiamide's effectiveness and safety profile to placebo to date. The significant finding of our study was that there was an improvement in symptoms of functional dyspepsia following the use of acotiamide compared to placebo. However, it could not reach statistical significance. Our results were similar to a previous meta-analysis done by Xiao et al. [11]. 

Improvement of major symptoms of bloating, postprandial fullness, and early satiety was higher in the acotiamide group than placebo. Tack et al. and Matsueda et al., which used weekly scores, both showed significant improvement in fullness, bloating, and satiety than placebo [6]. The seven-point Likert scale used in Kusunoki et al. [9] showed a symptom improvement rate of 31.6% in acotiamide compared to 16.7% in placebo. The Gastrointestinal Symptom Rating Scale (GSRS) questionnaire was used in Nakamura et al.; GSRS dyspepsia score and total GSRS score significantly improved in the acotiamide group.

All studies included in our meta-analysis used 100 mg acotiamide to compare its effects with placebo; Tack et al. [6] and Matsueda et al. [10] used 50 mg and 300 mg acotiamide in addition to standard 100 mg. One hundred mg showed more improvements than 50 mg and 300 mg in both studies compared to placebo, highlighting the importance of standard dosing regimens. Therefore, 100 mg acotiamide seems to be the appropriate dose in the treatment of FD. 

The analysis has also revealed an increase in gastric accommodation and gastric emptying time as compared to placebo. Kusunoki et al., Nakamura et al., and Tack et al. compared gastric accommodation rate and gastric emptying time of the drug to placebo [6,8,9]. Tack et al. [6], the only study was done in the European population, found no difference in gastric emptying rate between all groups of acotiamide and placebo; also, the gastric emptying rate did not improve following acotiamide administration. However, in a study by Kusunoki et al. and Nakamura et al., the gastric emptying rate and accommodation rate increased significantly in patients following acotiamide administration [8,9]. The difference could be due to different methods employed to measure gastric emptying. Tack et al. [6] measured gastric emptying using a 13-C urea breath test, solid diet, and with patients in sitting position. Meanwhile, Kusunoki et al. [9] resorted to using ultrasound, liquid meal, and patients in face-up position. A solid meal was used in Nakamura et al. [8], patients were in sitting position, and gastric scintigraphy was used.

The incidence of adverse events did not differ significantly between placebo groups and acotiamide groups in our analysis. Most of the adverse events were mild and of GI origin, like raised ALT, raised bilirubin, and raised serum prolactin. One study showed the incidence of biliary colic and angina pectoris; however, they were deemed unrelated or unlikely to be related to acotiamide [6]. Newer prokinetic drugs like tegaserod, itopride, cisapride, and mosapride have been used in randomized controlled trials for functional dyspepsia with some symptom improvement [12]. However, more studies are required, and a comparison of acotiamide with other prokinetic drugs may be helpful.

Limitations of our study

Most of the studies included in our meta-analysis were done in the Japanese population. Only one study by Tack et al. was carried out in Europe. More studies are required in other population groups to confirm the efficacy of acotiamide in other groups. As FD is a chronic recurring condition, relapse is common. Proper follow-up of patients has not been done in available studies to effectively aid in finding out the long-term benefits of acotiamide in addition to short-term relief. There are obvious limitations associated with the method of meta-analysis. There is a high chance of bias as well as the patients had to recall pretreatment symptom severity in some studies.

## Conclusions

Acotiamide is a promising therapeutic agent in the treatment of functional dyspepsia. There was some improvement in FD symptoms following acotiamide use compared to placebo, with no significant adverse effects. However, more studies need to be done in other population groups besides the Japanese population. In addition, follow-up of patients following treatment may be done to find out the drug's long-term benefits. RCTs comparing the efficacy of the drug with other newer prokinetic drugs may be helpful as well.
